# Multi-Sensor Fault Diagnosis of Underwater Thruster Propeller Based on Deep Learning

**DOI:** 10.3390/s21217187

**Published:** 2021-10-29

**Authors:** Chia-Ming Tsai, Chiao-Sheng Wang, Yu-Jen Chung, Yung-Da Sun, Jau-Woei Perng

**Affiliations:** 1 Department of Mechanical and Electro-Mechanical Engineering, National Sun Yat-sen University, Kaohsiung 804, Taiwan; d073020009@nsysu.edu.tw (C.-M.T.); d093020010@nsysu.edu.tw (C.-S.W.); 2 Naval Academy R.O.C., Kaohsiung 804, Taiwan; chungyj@cna.edu.tw; 3 Naval Meteorological and Oceanographic Office R.O.C., Kaohsiung 804, Taiwan; mrbig.g9114072005@gmail.com; 4 Department of Healthcare Administration and Medical Informatics, Kaohsiung Medical University, Kaohsiung 807, Taiwan

**Keywords:** propeller fault diagnosis, underwater thruster, deep learning

## Abstract

With the rapid development of unmanned surfaces and underwater vehicles, fault diagnoses for underwater thrusters are important to prevent sudden damage, which can cause huge losses. The propeller causes the most common type of thruster damage. Thus, it is important to monitor the propeller’s health reliably. This study proposes a fault diagnosis method for underwater thruster propellers. A deep convolutional neural network was proposed to monitor propeller conditions. A Hall element and hydrophone were used to obtain the current signal from the thruster and the sound signal in water, respectively. These raw data were fast Fourier transformed from the time domain to the frequency domain and used as the input to the neural network. The output of the neural network indicated the propeller’s health conditions. This study demonstrated the results of a single signal and the fusion of multiple signals in a neural network. The results showed that the multi-signal input had a higher accuracy than the one-signal input. With multi-signal inputs, training two types of signals with a separated neural network and then merging them at the end yielded the best results (99.88%), as compared to training two types of signals with a single neural network.

## 1. Introduction

Many unmanned marine robots (UMRs), such as unmanned surface vehicles and unmanned underwater vehicles, have been rapidly developed for marine research, including environmental monitoring (e.g., temperature, depth, and salinity data collection) [1], geological analysis of seabed rock formations and sediments, seabed topography [2], and underwater archeology [3]. As the application of UMRs is becoming widespread, safety and stability are important issues. Ou et al. [4] proposed a method for the fault diagnosis of ship propulsion systems. Data can be transmitted to a fault diagnosis center by international maritime satellites or the Internet of Things. Experts or expert system software can perform fault diagnoses and resolve problems.

For vehicles, the propulsion system is a kernel component. Damage to the propulsion system and loss of control may cause heavy losses. The health inspection of an underwater thruster is related to the normal operation of the propulsion system. Damage to the propeller is a common type of damage to underwater thrusters. When the propeller rotates at a high speed, a sharp decrease in the local water pressure forms vacuum bubbles. These subtle vapor bubbles generated by the cavitation phenomenon generate implosion and cause severe corrosion of the surface of the propeller. This phenomenon is called cavitation. As the thruster runs for a long time, the propeller is gradually damaged. Aktas et al. [5] used a hydrophone to collect sound in water and an underwater camera to obtain a cavitation video from two types of propellers. They used two types of signals to compare two different propellers and proved that the new propeller can reduce cavitation. When the propeller operates water for a long time, the ship performance may be reduced because the surface of the propeller is influenced by biofouling. Owen et al. [6] and Farkas et al. [7] used a computational fluid dynamics method to investigate the performance of a propeller influenced by biofouling. In addition to cavitation and biofouling, underwater noise caused by thrusters is another issue for marine animals. This type of low-frequency noise influences their behavior and causes pain. Sezen et al. [8] discussed the influence of biofouling roughness on cavitation and noise radiated underwater. When the propeller blade is ruptured or attached to an aquatic organism, an eccentricity effect occurs. The centrifugal force caused by the broken blade caused whirling vibrations in the shaft system. Ou et al. [9] proposed a method for diagnosing the shaft orbit. The finite element method was applied to obtain the time-domain waveform of the vibration signal and transfer it to obtain the shaft center orbit diagram.

Several machine learning methods have been proposed for fault diagnosis, including support vector machines [10,11,12,13], artificial neural networks (ANNs) [14], and deep learning [15]. Among these machine learning methods, the use of a deeper network structure has been proposed for deep learning, so that it can automatically learn useful features from big data and detect fault features. Many deep learning methods, such as stacked autoencoders [16], CNN [17], deep brief networks [18], long short-term memory (LSTM) [19,20], and recurrent neural networks (RNNs) [21], have been used to detect different faults. Lu et al. [22] proposed an enhanced CNN to detect in situ motor faults using vibration signals in an embedded system. Miao et al. [23] obtained the vibration signal from a gearbox and used an adaptive, densely connected convolutional autoencoder to detect faults in the gearbox. Eren et al. [24] used an adaptive one-dimensional (1D) CNN for bearing fault diagnosis, whereas Sun et al. [25] used a sparse deep-stacking network for bearing fault diagnosis.

A motor combined with a propeller provides propulsion power and can be used as the thruster of a vehicle. Thruster health is an important factor that supports safe navigation. The propulsion efficiency of the thruster may be reduced by a defective propeller. In unmanned aerial vehicle (UAV) applications, propeller damage may cause the UAV to drop to the ground and break. It also poses a human safety risk. Ghalamchi et al. [26] used accelerometer measurements and motor force commands as the inputs. A Kalman-filter-based method was proposed to evaluate the imbalance for each propeller. Iannace et al. [27] attached two paper tapes to the propeller surface to simulate an unbalanced propeller condition. The noise generated by the UAV was used to build a classification model using an ANN. The ANN model can then detect unbalanced blades using sound information. For USV applications, an artificial immune system and signal preprocessing were combined to detect faults in underwater thrusters [28]. With a single signal, the fault feature information may be insufficient in achieving an accurate fault classification. To solve the restriction from a single signal, a method based on multiple sensors was proposed to improve the accuracy of the results and the stability of the model. Nascimento and Valdenegro-Toro [29] used the control input, rotating speed, voltage, and current signals to build a nominal behavior model of an underwater thruster. The results showed that a multilayer perceptron with residuals achieved the best performance among several methods. Shao et al. [30] used a deep convolutional neural network (DCNN) for motor fault diagnosis. The current and vibration signals were transformed into the time frequency domain (TFD) using a wavelet transform. The DCNN was used to learn the features from the TFD image. A fully connected layer (FCN) was used to predict the motor conditions. Abed et al. [31] used different blade-breaking conditions to monitor a trolling motor. A discrete wavelet transform was used to extract the features from the current and vibration signals. An orthogonal fuzzy neighborhood discriminant analysis approach was used to reduce the feature redundancy. A time-delay neural network was designed for fault classification.

In addition to underwater thruster fault diagnosis [32,33,34], there are many fault diagnosis applications for marine robots [35]. Zhao et al. [36] used a particle filter to detect several fault conditions, including abnormal hydroacoustic positioning reference (HPR), loss of HPR, loss of Doppler velocity log (DVL), bias of DVL, and loss of thrust. Jiang et al. [37] proposed a diagnostic network combining a wide convolutional neural network and an extreme learning machine to detect actuator faults.

In this study, a DCNN model was proposed to detect fault propellers using multiple sensors. The results showed that the proposed method effectively diagnosed four types of propeller conditions.

The contributions of the present study are summarized below:

(1) Current, vibration, and voltage signals have been commonly used to accomplish fault diagnosis. In the present study, the proposed DCNN model diagnoses thruster propeller faults by extracting the current signal from a Hall element and the sound signal from a hydrophone. To the best of our knowledge, this study is the first to use a hydrophone to diagnose propeller faults through deep learning;
(2) Nascimento and Valdenegro-Toro [29] used an RNN model to detect propeller faults. The voltage, rotational speed, and current signals were used as the features. However, the highest accuracy of the results was only 78%. In comparison, the DCNN model proposed in this study achieved a 99.8% accuracy;
(3) Abed et al. [31] used a discrete wavelet transform to extract the features. An orthogonal fuzzy neighborhood discriminant analysis was used to select the best features as the input of the time-delayed neural network to compare the accuracy. However, this preprocessing flow is time-consuming. The present study used only the fast Fourier transform (FFT) to transform the signal from the time domain to the frequency domain. The DCNN had the ability to automatically learn features from the data, which significantly reduced the preprocessing time. The high accuracy of the results demonstrated excellent performance;
(4) This study proposed a multi-signal input for underwater thruster fault diagnosis using the DCNN model. The rotating speed of the thruster ranged from 2200 rpm forward to 2200 rpm reverse, at eight different speeds. Four conditions were proposed for the propeller: healthy, blade half-broken, blade fully broken, and biofouling simulated using silicon.

The remainder of this paper is organized as follows. Section 2 presents the fault conditions of the different propellers and the data collection flowchart. Section 3 describes the experimental data acquisition platform. The FFT was used to transform the data into the frequency domain. Different DCNN architectures were designed to train models using single and multiple signals. A visualization method was used to reduce the data from high to low dimensions. In Section 4, the features from different propeller conditions in the frequency domain are presented. The accuracies of the DCNN models using the different methods were compared. The model with the highest accuracy was used to determine the accuracy at different rotating speeds. The results demonstrate that separately training the two types of signals first and then merging them at the end can yield the best result among several methods. Section 5 presents the conclusions of the study.

## 2. Materials

**Figure 1:**
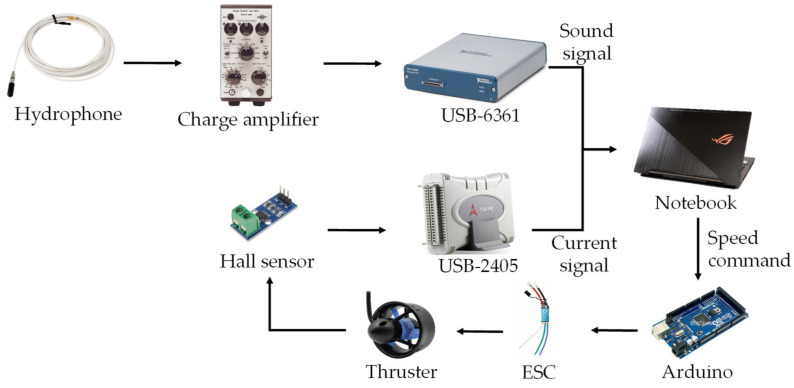
Architecture of the data collection platform.

Figure 1 shows the architecture of the data collection platform. The Arduino microcontroller was used to control the rotating speed of the Bluerobotics (2740 California St, Torrance, CA, USA) T200 thruster brushless motor with an electronic speed controller (ESC). The revolutions per minute (RPM) of T200 are approximately 3900 forward and 3800 reverse. Two types of signals, sound and current, were obtained using a hydrophone and a Hall device. The miniature hydrophone type 8103, manufactured by Brüel & Kjær (Nærum, Denmark), was used to obtain sound signals in water and transform them into electric signals. The frequency range of the hydrophone ranges from 0.1 Hz to 20 kHz. The voltage sensitivity of the cable at 20 °C was 80 µV/Pa. The charge amplifier 2635 manufactured by Brüel & Kjær could amplify the electric signal from the hydrophone. The data acquisition unit was USB-6361, developed by National Instruments (Austin, TX, USA). It provides a 16-bit analog-to-digital converter. The maximum sampling rate for a single channel was 2 MS/s. A Hall sensor (ACS711EX) was used to obtain the current signals. The Hall sensor allowed bidirectional currents from −31 A to +31 A, and the bandwidth was approximately 100 kHz. It provided high precision and reliability, with deviations ranging from 4% to −4%. The data acquisition unit was USB-2405, manufactured by ADLINK Technology(Taoyuan City, China). It provided a 24-bit high-performance dynamic signal capture USB model with four analog input channels, each of which providing 128 kS/s simultaneous sampling.

**Figure 2:**
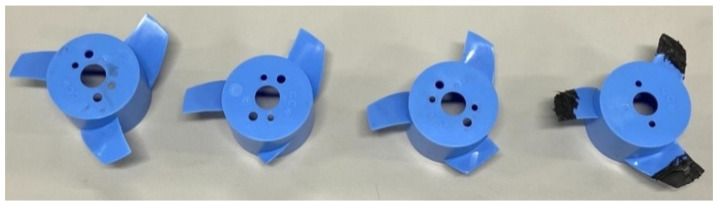
Different faults in blades. From right to left, the conditions are healthy, completely broken, half-broken, and simulated biofouling.

In Figure 2, four propellers made of polycarbonate were used to simulate different blade conditions. The diameter of the propeller was 76.2 mm. When the thruster is operated at a high rotating speed for a long time, the surface of the blade is gradually worn by impurities in water. If this abrasion accumulated for a long time, it may cause a sudden breakage of the blade, resulting in a half-broken or even completely broken blade. Another condition is biofouling; when a vessel has been mooring on water for a long time, some marine organisms, such as barnacles, algae, and sea oysters, attach to the surface of the blade. This obstruction increases the friction resistance and fuel consumption and reduces the propulsion power.

## 3. Methods

**Figure 3:**
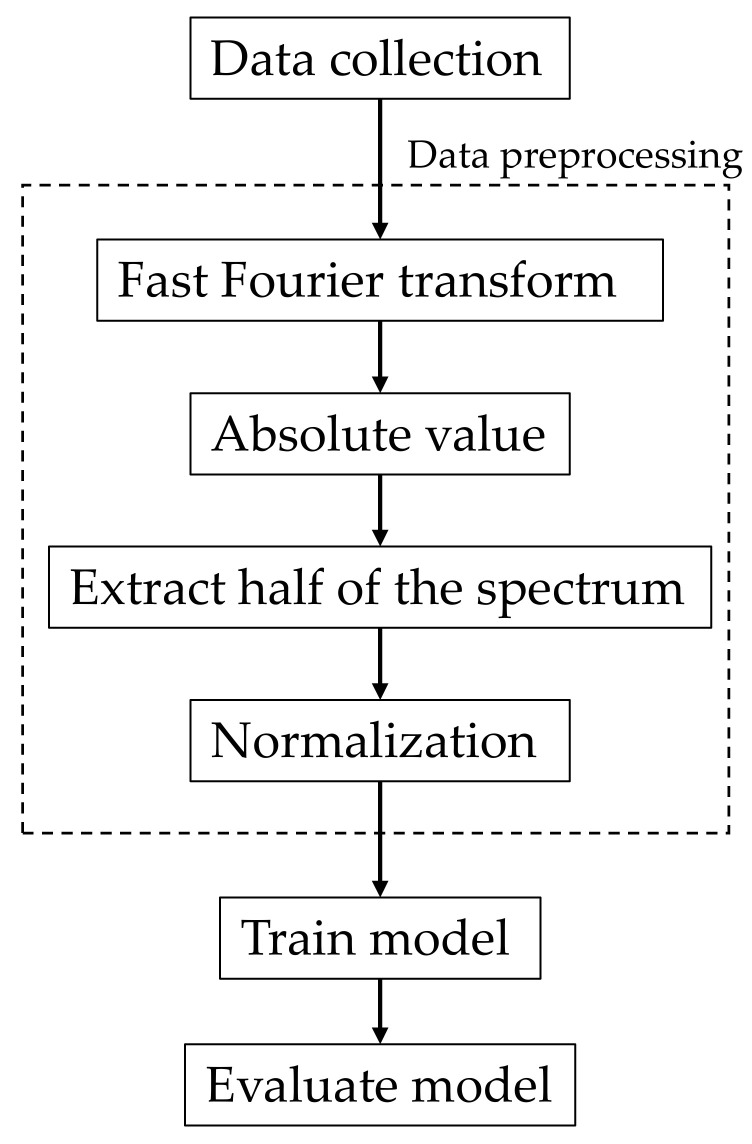
Flowchart of the underwater thruster fault diagnosis.

This study proposed an underwater thruster fault diagnosis technology using different types of sensors. A flowchart of the underwater thruster fault diagnosis is shown in Figure 3. First, the current and sound signals were collected under certain blade conditions on the thruster. Second, data preprocessing procedures were proposed to process the raw data. Third, the processed data were used as inputs for the proposed training model. Finally, the testing data were used to evaluate the accuracy of the proposed model.

### 3.1. Data Collection and Signal Preprocessing

#### 3.1.1. Data Collection

**Figure 4:**
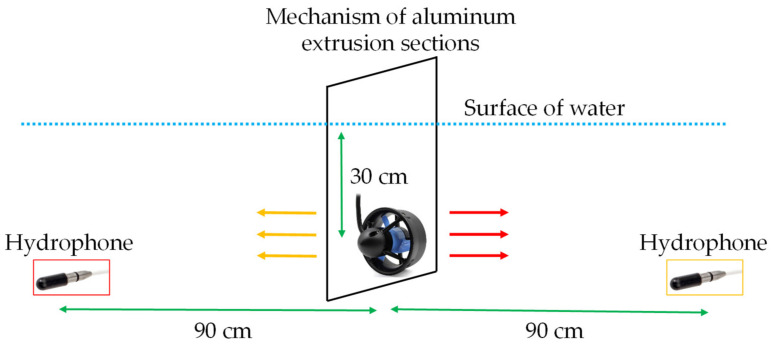
Thruster mounted on a mechanism of aluminum extrusion sections.

The propeller fault conditions are listed in Table 1. The thruster was mounted on an aluminum extrusion section, as shown in Figure 4. The distance between the surface of the water and the center of the thruster was approximately 30 cm. The hydrophone was placed opposite the water flow to prevent noise from influencing the sound signal. The distance between the hydrophone and thruster was 90 cm. The operating voltage of the thruster was set to 20 V. The thruster rotated between 2200 rpm forward to 2200 rpm reverse, which corresponded to a pulse-width modulation (PWM) range of 1700–1300. The PWM value between 1700 and 1300 was divided by 50 to obtain eight types of rotating speed data. When the thruster was operating, a Hall sensor and hydrophone were used to obtain data simultaneously. The current and sound signals were simultaneously collected by USB-6361 and USB-2405 at a sampling rate of 12,800 Hz. For each condition of the propeller, it included four fault conditions, eight rotating speeds, and 500 measurements, so that 16,000 (4 × 8 × 500) data points were collected for the current and sound data, respectively. These data were preprocessed first and then used as inputs for the proposed models. Due to the fact that the training was supervised learning, these data were required to create labels corresponding to the fault conditions. The output of the model is the classification results of the fault conditions. In this study, the proportions of training and test data were 80% and 20%, respectively.

#### 3.1.2. Data Preprocessing

**Figure 5:**
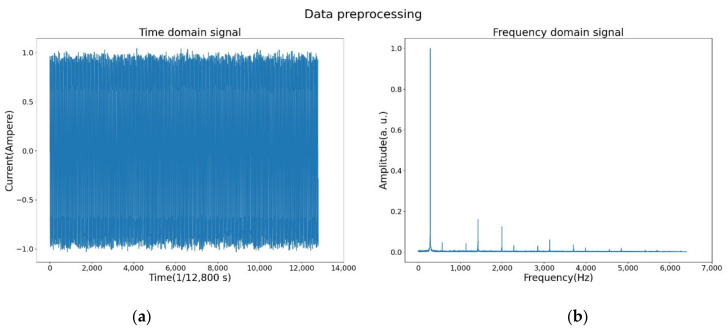
Transformation of a raw signal from the time domain to the frequency domain: (**a**) Current signal in time domain. (**b**) Current signal in frequency domain.

The raw data of the current and sound signals are one-dimensional time-domain signals. In the time domain, the signal intensity appeared to vary with time, and it was not easy to distinguish features, as shown in Figure 5a, which shows the variation in current data over 1 s. However, if the signal is transformed to the frequency domain, the features appear. All signals were a combination of a sine wave and a cosine wave, and FFT was used to transform the signals from the time domain to the frequency domain and to decompose it into sine waves with different amplitudes and frequencies. The FFT is expressed as follows:

(1) $$y\left(j\right)=\overset{n-1}{\underset{k=0}{\sum }}x\left(k\right)e^{-2ijk\frac{\pi }{n}},\;j=0,1,2,\ldots ,n-1$$

where $y\left(j\right)$ is the transformed signal. The data preprocessing is shown in Figure 3. The sampling rate was 12,800 Hz, and the sampling point was 12,800. After FFT, the signal data transformed to a complex plane and obtained the result a+bj, where a is the real part and bj is the imaginary part. The amplitude was calculated as $\sqrt{a^{2}+b^{2}}$. Due to the fact that the spectrum was symmetrical, half of the spectrum was extracted. Finally, normalization was used to map the data from 0 to 1, and these data were used as input to the DCNN. The signal transformed into the frequency domain is shown in Figure 5b. The signal strength was non-zero only at some specific frequencies, and these signals could be used as features to train the neural network (NN).

### 3.2. Deep Convolutional Neural Network

**Figure 6:**
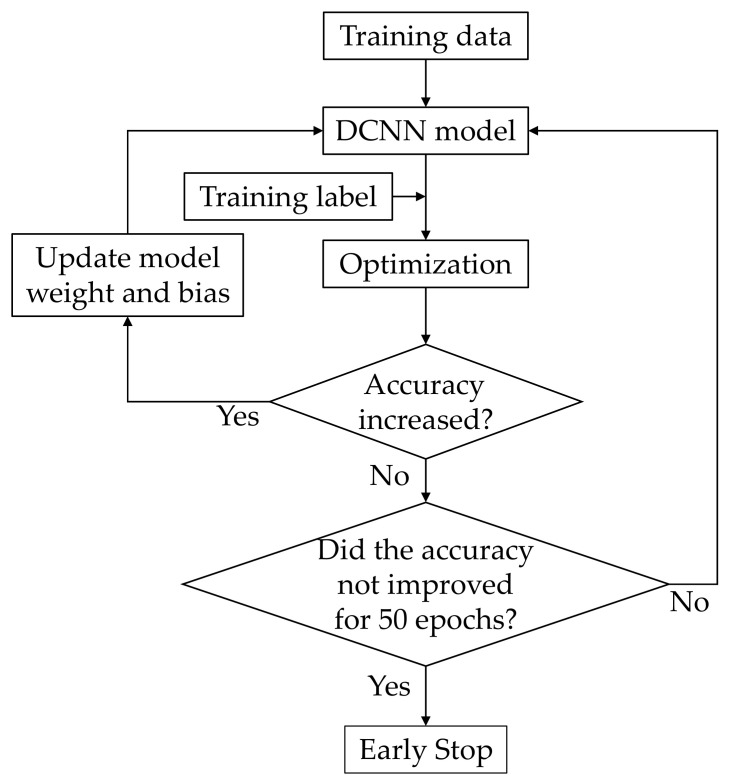
Training process of DCNN.

With the development of deep learning, DCNNs have shown excellent classification performance in many applications. The DCNN model can learn more features as the number of layers increases. The training process of the proposed model is shown in Figure 6. The preprocessed data were used as the input, and a DCNN model was designed to train the model. If the accuracy increased, the model weight and bias were saved, and training was continued until the accuracy failed to increase for more than 50 epochs.

The DCNN consisted of the following layers.

#### 3.2.1. Convolution Layer

The input of the current and sound signals was 1D data; hence, a 1D CNN was used. The kernel slid along the 1D data and obtained the feature map. The convolution computing formula for the 1D CNN is as follows:

(2) $$y_{i}=\sum_{m=1}^{n}w_{m}x_{i}+\beta ,$$

where $y_{i}$ is the convolutional neural output, $x_{i}$ is the value of the input, $w_{m}$ is the convolution weight of position m, m is the calculation count of the convolution filter, n is the total number of convolution filters, and β is the bias of the convolutional filter. In this study, the input signal had 6400 data points, the kernel size was 3, the stride was 1, and the activation function was ReLU. After the convolution, the width of the feature map became small. To maintain the shape of the feature map, zero padding was used.

#### 3.2.2. Maxpooling Layer

**Figure 7:**
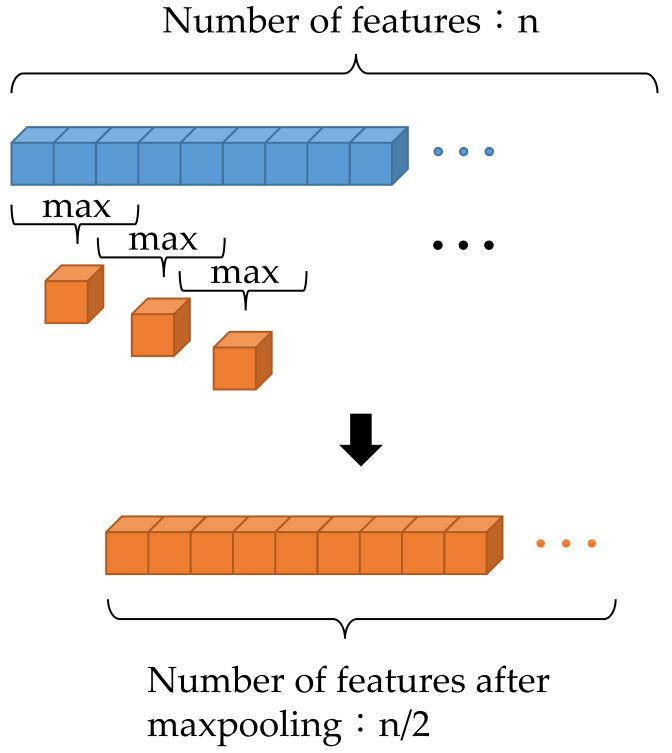
Schematic of maxpooling.

To reduce the quantity of data and reserve important features, maxpooling was applied after convolution. The maxpooling operation is shown in Figure 7. In maxpooling, the maximum number of features from the previous feature was chosen. In this study, the pool size was 3, the stride was 2, and zero padding was used, so that the width of the feature map after maxpooling was half of that before maxpooling.

#### 3.2.3. Global Maxpooling Layer

**Figure 8:**
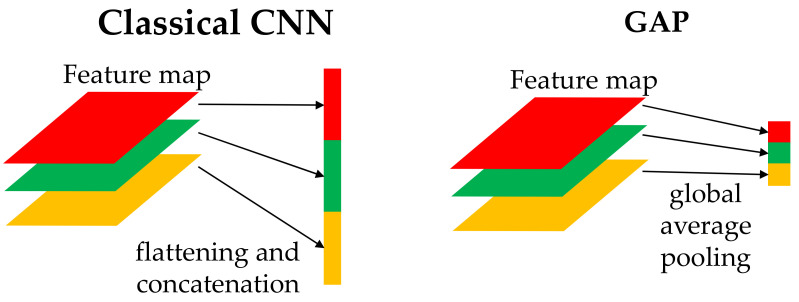
Comparison of FCN and GAP.

Global average pooling (GAP) was first proposed in Section 3.2 of Lin et al. [38]. This pooling operation was proposed to replace the fully connected layer (FCN) in a classical CNN. A comparison between the classical CNN and the GAP is shown in Figure 8. In a classical CNN, the last layer of the feature maps is flattened and connected to the FCN. The FCN was connected to the prediction layer, and an activation function was used to obtain the classification results. However, this method requires many tuning parameters and increases the training time. Occasionally, more than 80% of the parameters appeared in the last FCN. If a suitable regularizer is not used, overfitting may occur. For the GAP, the flattened step was replaced. Each layer of the last feature layer was averaged and concatenated, such that the output neuron from the GAP was the same as the number of feature maps. The GAP method greatly reduced the number of parameters and made the model more robust to avoid overfitting.

#### 3.2.4. Batch Normalization

The batch normalization method proposed by Google [39] was used in this study. When the CNN becomes deeper, the internal covariate shift causes the gradient to disappear during backpropagation. The batch normalization parameter normalizes the input value to resolve this problem. If there is a batch $B=\left\{x_{1},x_{2},x_{3},\ldots ,x_{m}\right\}$, the output $\left\{y_{i}\right\}$ after batch normalization can be calculated as follows:

(3) $$\frac{1}{m}\sum_{i=1}^{m}x_{i}\to \mu_{\beta },$$

(4) $$\frac{1}{m}\sum_{i=1}^{m}{\left(x_{i}-\mu_{\beta }\right)}^{2}\to \sigma_{\beta }{}^{2},$$

(5) $$\frac{x_{i}-\mu_{\beta }}{\sqrt{\sigma_{\beta }{}^{2}+\epsilon }}\to {\hat{x}}_{i},$$

(6) $$BN_{\gamma ,\beta }\left(x_{i}\right)=\gamma {\hat{x}}_{i}+\beta \to y_{i},$$

where m is batch size. For each batch, there were many elements, $x_{i}$; $\mu_{\beta }$ is the mean of the batch, $\sigma_{\beta }{}^{2}$ is the variance of the batch, ${\hat{x}}_{i}$ is the value after normalization, and ϵ is a small value 0.001 included to avoid a zero denominator. After normalization, the feature can be constricted in a range that is suitable for the DCNN to learn features. γ and β are the scale and shift learned by the NN, respectively. With this layer, the visualization results provided a good presentation.

#### 3.2.5. Dropout

**Figure 9:**
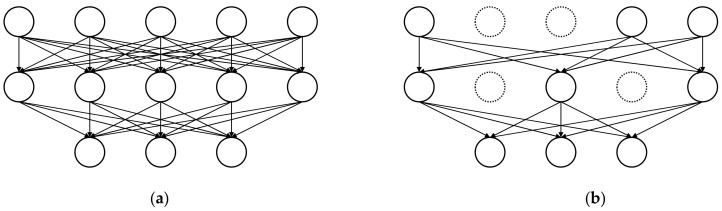
Comparison of neural networks with and without dropout. (**a**) Standard neural network, (**b**) Neural network with dropout.

Training a good model requires a large amount of data, as well as many parameters, of which, the latter may cause overfitting. To resolve this problem, dropout was proposed by Hinton [40] in 2012. In the same year, this method was used in AlexNet [41], which won the “ImageNet” image recognition competition. Since then, dropouts have been widely used in model training. An illustration of the dropout process is shown in Figure 9. Some neurons randomly stop working when this parameter is set. For example, if there are one thousand neurons in a layer and the parameter is set as 0.3, 300 neurons will be randomly chosen to stop working at an epoch.

The model should not be extremely sensitive to certain features. Although some features are lost, the model still needs to find other features from data to learn. The dropout method can reduce the coadaptation between neurons. The update of weights does not depend on some specific relation of neurons and avoids features that are only efficient in a special situation. When the relationship is broken, the NN learns other robust features to improve its accuracy.

#### 3.2.6. Prediction Layer

**Figure 10:**
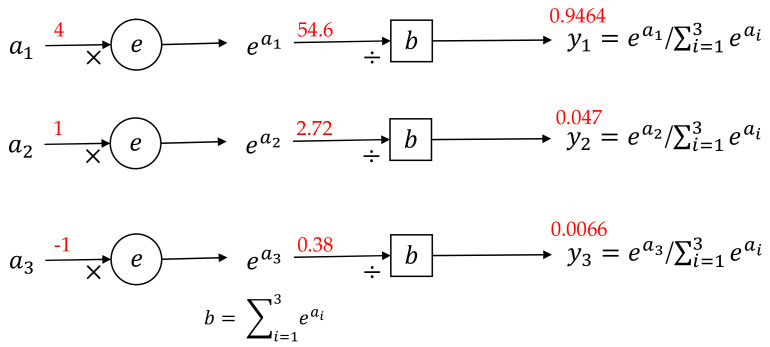
Calculation process of the softmax function.

A prediction layer was used to obtain the classification results after the GAP layer, batch normalization, and dropout. In this layer, the activation function softmax was used. An illustration of the softmax function calculation process is shown in Figure 10. The input value of the softmax function, $a_{i}$, increases exponentially to a new value. These values are added to obtain the sum result b, and are then divided by b. Through the softmax function, the input is mapped to a value between 0 and 1, which can be perceived as the probability of every classification result. The probability with the largest value was chosen as the prediction class.

### 3.3. Multi-Sensor Fusion

**Figure 11:**
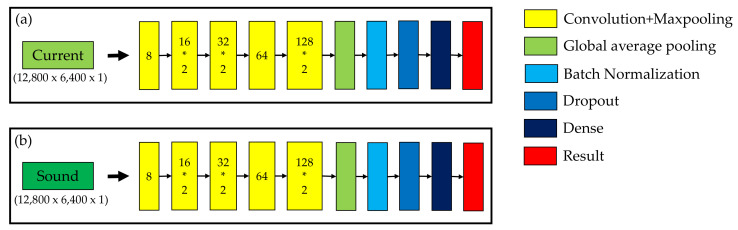
Single signal training model: (**a**) current signal; (**b**) sound signal.

**Figure 12:**
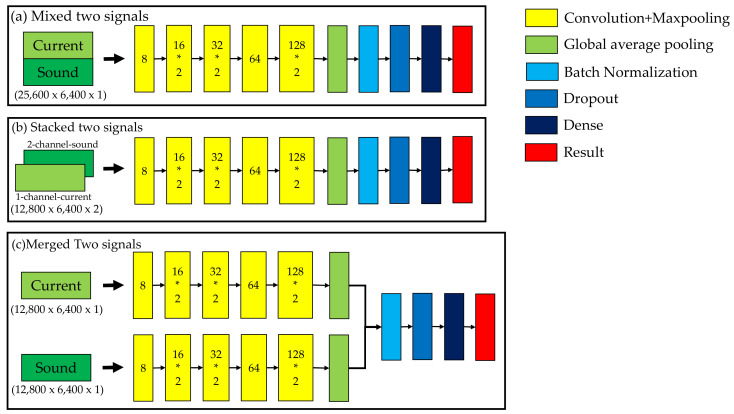
Multi-signal training model.

In this study, two types of data types, current and sound, were used to analyze the propeller conditions. Each type of signal included 12,800 data points, and each data point included 6400 measurements. Single and multiple signals were proposed to compare accuracy. In Figure 11, the current and sound signals are trained separately to demonstrate the results for a single signal. In Figure 12, different combination methods of the two signals are proposed to compare the results. In Figure 12a, two signals are mixed to train the model. Each type of signal included 12,800 data points; therefore, the total number of data points was 25,600. In Figure 12b, two signals are stacked to train the model. The first and second channels are the current and sound signals, respectively. Thus, the third dimension is 2. In Figure 12c, two independent signals were trained separately first, and, after the global average pooling, these two signals were merged and used together to predict the results.

### 3.4. t-Distributed Stochastic Neighbor Embedding (t-SNE) Algorithm

For 1D, 2D, and 3D data, it is easy to visualize and provide intuitive results for researchers to analyze. However, deep learning usually involves high-dimensional data, and dimensionality reduction methods, such as decision tree [42], random forest [43], and principal component analysis (PCA) [44], can be used to visualize these data. The PCA method can use a few linear combinations to present the most information on the origin data. The main information of the origin variable can be saved and simplified. Every principal component is a linear combination of the origin variable and a substantially reduced origin variable. However, PCA cannot deal with the local structure of a dataset. To solve this problem, t-SNE was designed [45]. The goal of t-SNE is to transform high-dimensional data into low-dimensional data. This is an improvement over SNE [46]. The t-SNE algorithm chooses points in high dimensions and determines the projection of these points in low dimensions.

**Figure 13:**
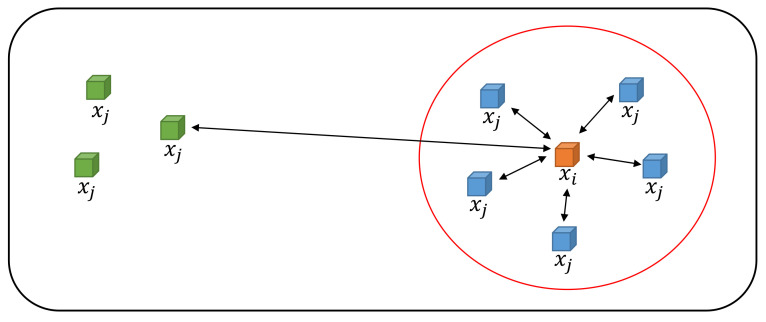
Illustration of data with different Euclidean distances.

In Figure 13, there is a dataset $D=\left\{x_{1},x_{2},x_{3},\;\ldots x_{N}\right\}$. The Euclidean distance, $d\left(x_{i},x_{j}\right)=∥x_{i}-x_{j}∥$, is used to calculate the distance between a pair of points $x_{i}$ and $x_{j}$. For a high dimension, $x_{i}$ and $x_{j}$ are a pair of close points. $x_{i}$ is the Gaussian distribution center with variance $\sigma_{i}$. $p_{j|i}$ is the similarity score indicating how close $x_{j}$ is to $x_{i}$, and it is given by the following formula: if two points are close, $p_{j|i}$ is large, and vice versa.

(7) $$p_{j|i}=\frac{\exp\left(-{∥x_{i}-x_{j}∥}^{2}/2\sigma_{i}^{2}\right)}{\sum_{k\neq i}\exp\left(-{∥x_{i}-x_{k}∥}^{2}/2\sigma_{i}^{2}\right)},\;p_{i|i}=0$$

In SNE, although the Euclidean distances of $x_{i}$ and $x_{j}$ are the same, $p_{i|j}$ is not equal to $p_{j|i}$, because the σ in each is unique. If $x_{i}$ is in the dense region, a small variance value is chosen, and vice versa. A pair of points $x_{i}$ and $x_{j}$ will appear, where one is in a dense region and the other is in a relatively sparse region, enabling the variance value to be distinguished. To resolve this problem, the joint probability, which is the symmetrized conditional probabilities in t-SNE, is expressed as follows to make $p_{ij}=p_{ji}$ and used as the final similarity score in high dimensions.

(8) $$p_{ij}=\frac{p_{i|j}+p_{j|i}}{2N},$$

where $p_{i|j}$ is the similarity score with center $x_{j}$, $p_{j|i}$ is the similarity score with center $x_{i}$, and N is the number of data points. If two points are close in the high-dimensional origin space, the value of $p_{ij}$ will be large.

For high dimensions, the points can be explained as a normal distribution. In low dimensions, if singularities exist, the Gaussian distribution will be more sensitive, deviate from the location with most points, and have a larger variance. To address this problem, Student’s *t*-distribution was used because it has a higher tail, is not sensitive to singularities, and is more robust. This ensures that the crowd points in the low dimension can effectively group. The joint probability in low dimensions can be calculated as follows:

(9) $$q_{ij}=\frac{{\left(1+{∥y_{i}-y_{j}∥}^{2}\right)}^{-1}}{\sum_{k\neq l}{\left(1+{∥y_{k}-y_{l}∥}^{2}\right)}^{-1}},$$

where $y_{i}$ and $y_{j}$ are a pair of close points, and $y_{i}$ is the center of the Student’s *t*-distribution. A Student’s *t*-distribution with one degree of freedom is used in low dimensions because, in the low-dimensionality map, ${\left(1+{∥y_{i}-y_{j}∥}^{2}\right)}^{-1}$ approaches an inverse square law for large pairwise distances $∥y_{i}-y_{j}∥$.

The main goal of t-SNE is to make $q_{ij}$ in low dimensions reflect the probability $p_{ij}$, at high dimensions. This implies that a two-dimensional map has a similar structure. To measure the difference between these space probability distributions, the Kullback–Leibler divergence was used as follows:

(10) $$KL\left(P∥Q\right)=\sum_{i}\sum_{j\neq i}p_{ij}log\frac{p_{ij}}{q_{ij}},\;p_{ii}=q_{ii}=0$$

The Kullback–Leibler divergence was used as the cost function, and the gradient descent algorithm was applied. It minimizes the relative positions of the data between two data points to preserve the local structure of the data. The gradient of the Kullback–Leibler divergence between $p_{ij}$ and $q_{ij}$ is given by

(11) $$\frac{\delta C}{\delta y_{i}}=4\underset{j}{\sum }\left(p_{ij}-q_{ij}\right)\left(y_{i}-y_{j}\right){\left(1+{∥y_{i}-y_{j}∥}^{2}\right)}^{-1},$$

## 4. Results

**Figure 14:**
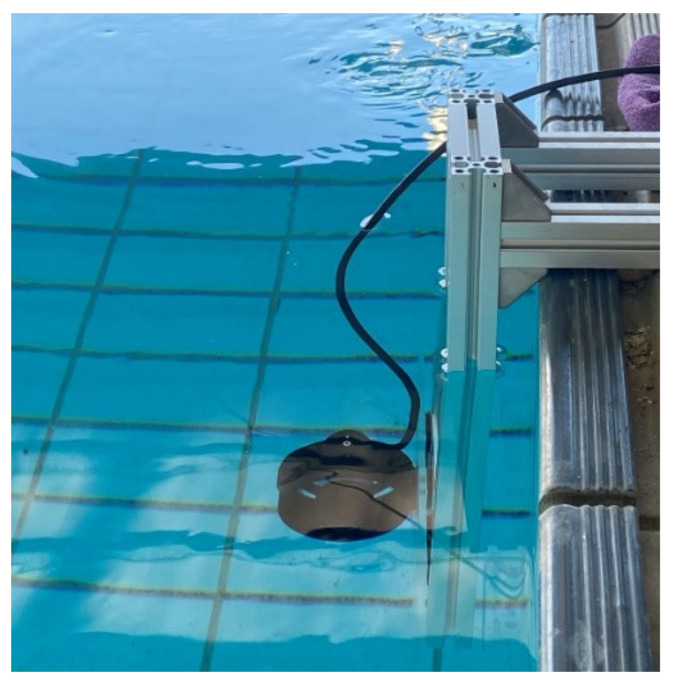
Experimental mechanism in water.

In this study, the experimental field was a swimming pool. The mechanism used to mount the thruster is shown in Figure 14. Multiple sensors, a Hall element, and a hydrophone were used to obtain the current and sound signals. The data were acquired at multiple rotation speeds. The accuracies of the proposed methods were compared to determine the best solution for thruster propeller fault diagnosis. To visualize the result, the t-SNE algorithm [45] was used to reduce the dimensionality of the feature map.

### 4.1. Data Preprocessing Results

**Figure 15:**
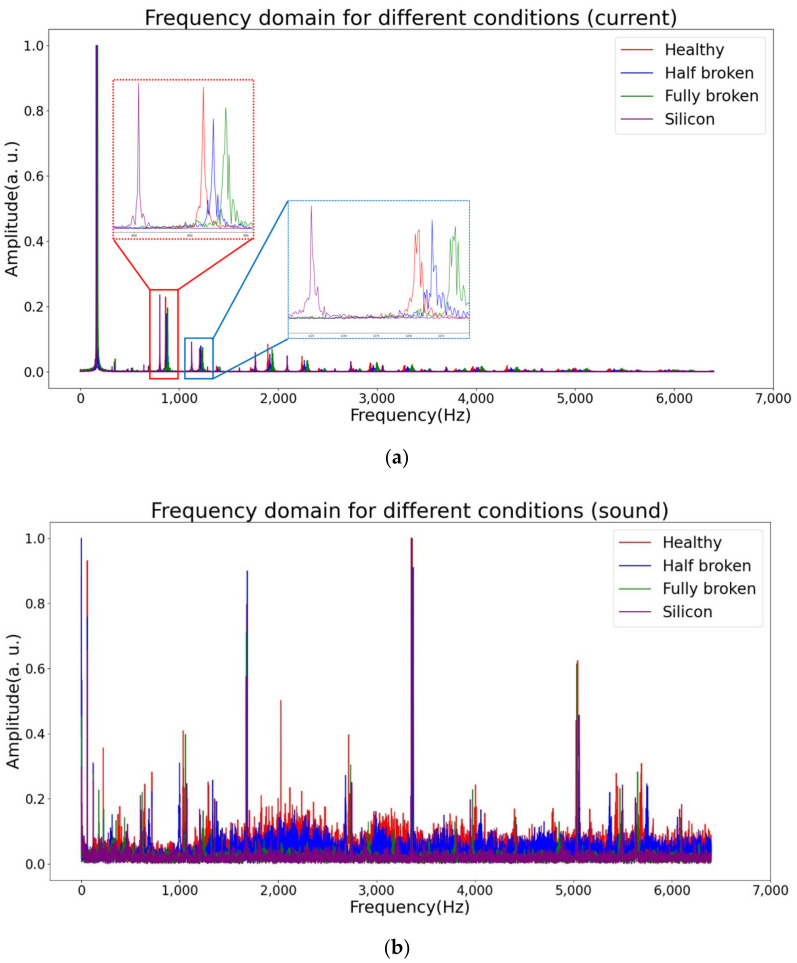
Signals for different propeller conditions on the frequency domain: (**a**) current signal; (**b**) sound signal.

The thruster was equipped with different propellers to collect the current and sound signals. However, the signals obtained from the sensors were in the time domain, and it was difficult to observe the features directly. To compare the signal features among the different propeller conditions, FFT was used to transform the signals to the frequency domain. The transformation results are shown in Figure 15. The rotational speed of the thruster was 1273 rpm. The FFT results from the different propellers were overlapped to observe the difference.

Figure 15a shows the FFT results from the current signals, whereas Figure 15b shows the FFT results from the sound signals. The red signals are for the healthy propeller condition, the blue signals are for the half-broken propeller condition, the green signals are for the fully broken propeller condition, and the purple signals are for the propeller with silicon. The main frequencies of the four conditions were similar for the current signals in Figure 15a. However, for harmonic frequencies, half-broken and fully broken features exist at similar frequencies and have similar amplitudes. The propeller with silicon is obviously different from the others. This phenomenon may be caused by an increase in water resistance. The silicon on the surface of the blade increased the resistance when the thruster rotated. For the sound signal in Figure 15b, it can be observed that every condition seems to be disorganized. However, there is still a slight difference in the amplitudes of the different conditions. As a result of the strong learning ability of DCNN, it can learn features from these slight clues.

### 4.2. Classification Results

To evaluate the results of the different methods, the following formulas were used to calculate the average accuracy of the different methods:

(12) $$Acc_{xxxx}=\frac{T_{xxxx}}{T_{xxxx}+F_{xxxx}}\times 100\%$$

(13) $$Acc_{mode}=\frac{Acc_{1300}+Acc_{1350}+Acc_{1400}+Acc_{1450}+Acc_{1550}+Acc_{1600}+Acc_{1650}+Acc_{1700}}{8}$$

(14) $$Acc_{method}=\frac{Acc_{hl}+Acc_{hb}+Acc_{fb}+Acc_{si}}{4}$$

where $Acc_{xxxx}$ denotes the accuracy at different rotating speeds. For each rotating speed, there were 100 testing data points. $Acc_{mode}$ denotes the average accuracy of eight types of rotating speed commands for one type of propeller condition. For each propeller condition, 800 testing data points were used. $Acc_{method}$ indicates the average accuracy of the different methods. For each method, 3200 testing data points were used. $Acc_{hl},\;Acc_{hb},\;Acc_{fb},$ and $Acc_{si}$ indicate the healthy propeller, half-broken propeller, fully broken propeller, and propeller covered with silicon, respectively. The quantity of testing data mentioned above is for a single signal. For two signals, the quantity of testing data is double this quantity.

Table 2 presents the accuracy for different propeller conditions and the average accuracies of the different methods. Sensor fusion yields better results than a single signal. The model with the current signal provided lower accuracy for the half-broken and fully broken conditions. The model with the sound signal provided lower accuracy in the half-broken condition. However, among the proposed methods of two signals, the average accuracies of the stacking and merging methods were over 99% for these conditions, and the accuracy of the merging method was much higher. This confirmed that multiple signals with the merging method yielded the best results.

In Table 3, the method with the highest accuracy, the merging of two signals, was used to present the accuracy of different rotating commands for each propeller condition. An accuracy of 100% was achieved under most conditions, except for a few. This result demonstrated that, compared with a single signal, multiple signals with a merged structure yielded a better performance.

**Figure 16:**
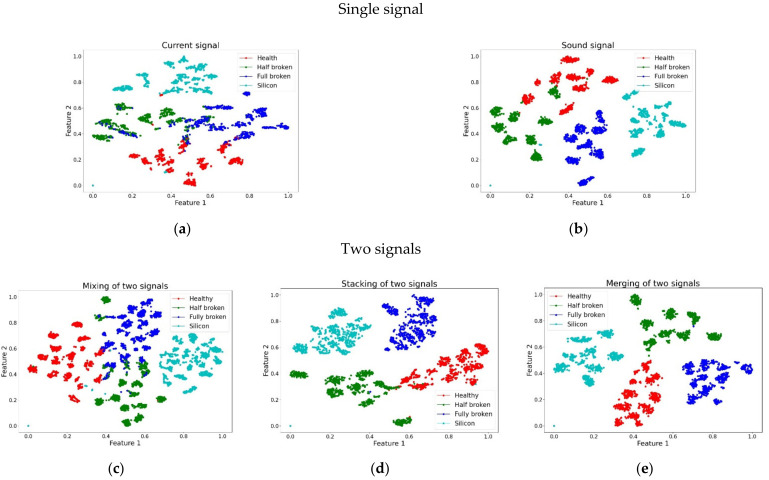
Visualization results of feature vectors for different methods: (**a**) current signal; (**b**) sound signal; (**c**) mixing of two signals; (**d**) stacking of two signals; (**e**) merging of two signals.

Only the accuracy value was expressed for the methods mentioned above, and the features of high-dimensional data could not be observed intuitively. To visualize the features learned by the CNN, the t-SNE algorithm [45] was utilized to reduce the features. It is a dimensionality reduction method that reduces multi-dimensional feature vectors to two-dimensional feature vectors. The visualization results for the feature vectors are shown in Figure 16. According to the visualization results, the four propeller conditions are presented in different colors. Each color can be divided into eight small regions corresponding to eight rotational speeds. For a single signal, as shown in Figure 16a, b, it can be observed that some points for different propeller conditions overlap with other conditions. Therefore, the accuracy of a single signal is lower. For two signals, as shown in Figure 16c–e, only a few features overlap with other features. Figure 16e shows the most clearly separated features under different conditions.

### 4.3. Prediction Time with Different Methods

To evaluate the efficiency of the different methods, the prediction times for 100 data points are listed in Table 4. The prediction time was the average of 100 data points. The variation in the prediction times for the different methods was very small. The cost times of the stacking and merging methods were slightly higher than those of the other methods. However, these two methods also provided a higher accuracy. The prediction time of 0.0198 s of the merging method was fast enough for fault diagnosis, and this method yielded the highest accuracy.

### 4.4. Calculation System

An i7-7700k central processing unit manufactured by Intel^®^ was used. A GeForce^®^ GTX 1080 Ti graphics processing unit manufactured by NVIDIA was used to execute the large matrix calculations. The PC had 16 GB of random access memory to save large datasets. The science data processing language, Python, was used because of its strong development tools and libraries, such as keras, tensorflow, and scikit-learn.

## 5. Conclusions

For a thruster in water, it is difficult to quickly identify abnormal propellers. Thus, propellers can cause significant damage. Regardless of whether the vessel is manned, it is important to have the capability to self-diagnose thruster faults. In addition, the fault diagnosis technology can be used not only on the thruster, but also on other equipment, such as the gear box, generator, or bearing. With appropriate sensors, irregular signals can be collected to develop fault diagnosis applications. The equipment usually has some special irregularities before failure. Sensors can be set up in places where failures are more likely to occur. If some features of a fault appeared and were detected, the vessel could immediately return in order to repair. Other vessels could be arranged to transfer goods to avoid schedule delays. The manufacture of a customized propeller could begin and reduce the idle period of the vessel.

This study proposed a method for the fault diagnosis of underwater thruster propellers. The experimental conditions included multiple rotating speeds (2200 rpm forward to 2200 rpm reverse), multiple sensors (current and sound signals), and multiple propeller conditions (healthy, half-broken, fully broken, and silicon-attached). A single signal and two signals with different data combination methods were used to compare the accuracy and efficiency of the fault diagnosis. The results demonstrated that the merging of the current and sound signals provided the best result (99.88%) among the studied methods.

The experimental location of this study was a swimming pool, which is an ideal environment for acquiring data. Although some sound noise is caused by the drainage hole at the pool bottom, the noise is not as significant as the actual sea. In the future, thrusters with different propeller fault conditions will be equipped on a USV to acquire current and sound signals in the actual sea. Furthermore, the DCNN model will be used and revised for real-world applications.

## Figures and Tables

**Table 1:** Types of propeller blade conditions.

Propeller Number	Condition
1	Healthy blade
2	Half-broken blade
3	Fully broken blade
4	Silicon-attached blade

**Table 2:** Accuracy of different methods under different conditions.

Method	Accuracy for Different Conditions				
	Healthy	Half-Broken	Fully Broken	Silicon	Average
Current signal	96.25%	90.38%	87.63%	99.00%	93.32%
Sound signal	98.50%	86.88%	99.75%	99.75%	96.22%
Mixing of two signals	99.06%	97.56%	95.00%	99.56%	97.80%
Stacking of two signals	99.25%	99.00%	99.75%	99.88%	99.47%
Merging of two signals	100%	99.75%	99.75%	100%	99.88%

**Table 3:** Accuracy of the method of merging two signals under different conditions and rotating speeds.

Propeller Condition	Rotating Command								
	1300	1350	1400	1450	1550	1600	1650	1700	Average
Healthy	100%	100%	100%	100%	100%	100%	100%	100%	100%
Half-broken	100%	100%	100%	100%	98%	100%	100%	100%	99.75%
Fully broken	100%	100%	100%	100%	100%	98%	100%	100%	99.75%
Silicon	100%	100%	100%	100%	100%	100%	100%	100%	100%
							Total average		99.88%

**Table 4:** Predict time of different methods.

Method	Prediction Time(s) for 100 Data Points
Current signal	0.0117
Sound signal	0.0114
Mixing of two signals	0.0116
Stacking of two signals	0.0161
Merging of two signals	0.0198

## Abbreviations

The following abbreviations are used in this manuscript:

Data preprocessing:	
$y\left(j\right)$	Transformed signal after FFT
Convolution Layer:	
$x_{i}$	Input of convolutional neural
$w_{m}$	Convolution weight
m	Calculation count of the convolution filter
β	Bias of the convolutional filter
n	Total number of convolution filters
$y_{i}$	Convolutional neural output
Batch Normalization:	
m	Batch size
$x_{i}$	Every element for each batch
$\mu_{\beta }$	Mean of the batch
${\sigma_{\beta }}^{2}$	Variance of the batch
${\hat{x}}_{i}$	Value after normalization
ϵ	Small value to avoid a zero denominator
γ	Scale learned by the NN
β	Shift learned by the NN
$y_{i}$	Output after batch normalization
t-Distributed Stochastic Neighbor Embedding (t-SNE) Algorithm:	
D	A data set $D=\left\{x_{1},x_{2},x_{3},\;\ldots x_{N}\right\}$
$d\left(x_{i},x_{j}\right)$	Euclidean distance between a pair of points $x_{i}$ and $x_{j}$
$x_{i}$	Gaussian distribution center with variance $\sigma_{i}$
$x_{j}$	Other points
$p_{i|j}$	Similarity score with center $x_{j}$
$p_{j|i}$	Similarity score with center $x_{i}$
N	Number of data points
$\sigma_{i}$	Variance
$y_{i}$	Center of the Student’s *t*-distribution
$y_{j}$	Other points
KL	Kullback–Leibler divergence
Other abbreviations:	
UMR	Unmanned marine robots
ANN	Artificial neural networks
LSTM	Long short-term memory
RNN	Recurrent neural networks
1D	One-dimensional
UAV	Unmanned aerial vehicle
DCNN	Deep convolution neural network
TFD	Time frequency domain
FCN	Fully connected layer
HPR	Hydroacoustic positioning reference
DVL	Doppler velocity log
FFT	Fast Fourier transform
ESC	Electronic speed controller
RPM	Revolutions per minute
PWM	Pulse-width modulation
NN	Neural network
GAP	Global average pooling
FCN	Fully connected layer
t-SNE	t-Distributed stochastic neighbor embedding
PCA	Principal component analysis
